# X-ray diffraction analysis of matter taking into account the second harmonic in the scattering of powerful ultrashort pulses of an electromagnetic field

**DOI:** 10.1038/s41598-021-83183-3

**Published:** 2021-02-11

**Authors:** M. K. Eseev, A. A. Goshev, K. A. Makarova, D. N. Makarov

**Affiliations:** Northern (Arctic) Federal University, Northern Dvina 17, Arkhangelsk, Russia 163002

**Keywords:** X-rays, Ultrafast lasers, High-harmonic generation

## Abstract

It is well known that the scattering of ultrashort pulses (USPs) of an electromagnetic field in the X-ray frequency range can be used in diffraction analysis. When such USPs are scattered by various polyatomic objects, a diffraction pattern appears from which the structure of the object can be determined. Today, there is a technical possibility of creating powerful USP sources and the analysis of the scattering spectra of such pulses is a high-precision instrument for studying the structure of matter. As a rule, such scattering occurs at a frequency close to the carrier frequency of the incident USP. In this work, it is shown that for high-power USPs, where the magnetic component of USPs cannot be neglected, scattering at the second harmonic appears. The scattering of USPs by the second harmonic has a characteristic diffraction pattern which can be used to judge the structure of the scattering object; combining the scattering spectra at the first and second harmonics therefore greatly enhances the diffraction analysis of matter. Scattering spectra at the first and second harmonics are shown for various polyatomic objects: examples considered are 2D and 3D materials such as graphene, carbon nanotubes, and hybrid structures consisting of nanotubes. The theory developed in this work can be applied to various multivolume objects and is quite simple for X-ray structural analysis, because it is based on analytical expressions.

## Introduction

X-ray diffraction (XRD) analysis of matter, as well as X-ray crystallography (XRC) are the most powerful methods for determining the structure of matter^1–5^. Most crystal structures and many molecules were discovered by these methods. They underlie many modern discoveries in the fields of physics, chemistry, biology, medicine and crystallography, e.g.^2,6–10^. Currently, these basic methods of X-ray structural analysis are supplemented and expanded in connection with the creation of new types of radiation sources, small sizes of objects under study, etc. As a result, separate directions in X-ray structural analysis appear, for example, grazing-incidence small-angle scattering (GISAS), transmission small-angle scattering (SAS), grazing-incidence diffraction (GID), diffuse reflectometry (DR), single crystal monochromatic diffraction (SXD), powder diffraction (EDX, ADX), fiber diffraction (FD) and others^2,3,5,11,12^. The theoretical basis of these methods has been understood for some time^1,3^; their physical interpretation is based on X-ray diffraction from various polyatomic structures. By analyzing the diffraction pattern, the structure of the substance can be judged. Usually, the theory of X-ray diffraction by various periodic and complex structures is described as the scattering of plane waves of infinite duration in time^1,3^. However, the processes of scattering of high-power ultrashort pulses (USPs) with femto- and especially attosecond time resolution by structures of this kind have been little studied so far. Moreover, at present, powerful USP sources are being introduced, for example, the European X-ray Free Electron Laser (XFEL), the Linear Accelerator of a Coherent Light Source (LCLS), and the Free Electron laser Radiation for Multidisciplinary Investigations (FERMI), all of which now enable this kind of research to be carried out.

In the last two decades, the generation of isolated attosecond pulses through high harmonic generation has provided a powerful tool for studying many important physical processes on the attosecond timescale^13^. Indeed, there is a tendency towards an increase in the power of USPs of an electromagnetic field and a reduction in their duration^14–16^. Research is actively being carried out, the technique of X-ray free electron lasers (e.g. XFEL) is being improved^16^, and a subfemtosecond barrier with a high peak power has also been achieved, which makes it possible to study excitation in a molecular system, and the motion of valence electrons with high temporal and spatial resolution^17^.

Studies of the structure and composition of nanosystems and biomolecules using USP scattering are possible for systems in both stationary and nonstationary states. Nonstationary objects can be studied only with the use of USPs, since the characteristic time in such systems is less than, or comparable to, the femtosecond timescale. Such processes can be the formation and rearrangement of chemical bonds, intra-atomic and molecular processes, etc.^18–22^. For example, the use of nanosystems in various devices is primarily associated with the charge transfer process, i.e. on changes in states over time^18^. It is for this reason that considerable interest is shown in real-time observations of electron dynamic processes.

The theoretical basis for carrying out this kind of research is rather poorly developed and often does not take into account the aspects of scattered USPs^14,16,23–28^, which is why theoretical studies of the aspects of USPs in their scattering by various polyatomic systems are currently being carried out. In spite of this, the magnetic component of such USPs is usually not taken into account. Indeed, it is well known that the force acting on a charged particle from the magnetic component of the electromagnetic field is approximately *c* = 137 (au) times less than its electrical component. However, when considering some phenomena, its contribution is very significant and neglecting it can lead to a misunderstanding of certain processes. For example, in^29^, when considering tunneling ionization in a strong field regime, its contribution was found to be very significant. In^30^, taking into account the magnetic component of USPs led to the possibility of detecting the orientation of molecular anions in space; and in^28^, it was also shown that the magnetic component must be taken into account in the scattering of USPs by various polyatomic systems.

It was found that taking into account the magnetic component of a USP leads to the appearance of the second harmonic. A joint analysis of the spectra of USP scattering at the fundamental and second harmonics provides more information about the object under study. Objects such as graphene, carbon nanotubes (CNTs), and a hybrid structure consisting of CNTs are considered as examples. We will use the atomic system of units where $\hbar = 1,|e| = 1,m_{e} = 1$.

## Theoretical basis

Consider an arbitrary polyatomic system on which a USP is falling in the direction $${\mathbf{n}}_{0}$$. The duration of such a pulse will be assumed to be much less than the characteristic atomic time $$\tau_{a} \sim 1$$, i.e.$${\raise0.7ex\hbox{$\tau $} \!\mathord{\left/ {\vphantom {\tau {\tau_{a} }}}\right.\kern-\nulldelimiterspace} \!\lower0.7ex\hbox{${\tau_{a} }$}} \ll 1$$. This will allow us to use the sudden disturbance approximation, in which the intrinsic Hamiltonian of the system can be neglected, since the electron in the atom does not have time to evolve under the action of the atomic field^26^. It should be added that the condition $${\raise0.7ex\hbox{$\tau $} \!\mathord{\left/ {\vphantom {\tau {\tau_{a} }}}\right.\kern-\nulldelimiterspace} \!\lower0.7ex\hbox{${\tau_{a} }$}} \ll 1$$ for using our approximation is not strict, and it is sufficient to assume, as shown in^26^, that $$\omega_{0} \tau_{a} \gg 1$$, where $$\omega_{0}$$ is the carrier frequency of the falling USP. Obviously, the condition $$\omega_{0} \tau_{a} \gg 1$$ is satisfied for X-ray frequencies. Let us consider the intensity of the electromagnetic field of a USP in a general form, i.e. it will be assumed to depend on coordinates and time $${\mathbf{E}}({\mathbf{r}},t)$$. We are interested in electromagnetic fields that are not strong enough to allow for relativistic effects. As obtained in^28^ (see also^26,27^), taking these approximations into account, the probability $$W$$ of a photon being created of a given frequency $$\omega$$ per unit solid angle $$\Omega_{{\mathbf{k}}}$$ with a simultaneous transition of a polyatomic system from the main state to all possible final states (hereinafter, the scattering spectrum of USPs) is determined:

1 $$\frac{{d^{2} W}}{{d\omega d\Omega_{{\mathbf{k}}} }} = \frac{1}{{\left( {2\pi } \right)^{2} }}\frac{1}{{c^{3} \omega }}\left( {N_{a} N_{e} S(\omega ,{\mathbf{n}},{\mathbf{n}}_{{\mathbf{0}}} ) + \delta_{N} ({\mathbf{p}})N^2_{e} F(\omega ,{\mathbf{n}},{\mathbf{n}}_{{\mathbf{0}}} )} \right),$$

where $S(\omega ,{\mathbf{n}},{\mathbf{n}}_{{\mathbf{0}}} )=G(\omega ,{\mathbf{n}},{\mathbf{n}}_{{\mathbf{0}}} )-F(\omega ,{\mathbf{n}},{\mathbf{n}}_{{\mathbf{0}}} )$, *N*_*a*_ is the total number of atoms in the system, $N_{e}$ is the number of the electrons in an atom, *c* is the speed of light, ${\mathbf{n}}$ is the direction of the created photon emission, $G(\omega ,{\mathbf{n}},{\mathbf{n}}_{{\mathbf{0}}} )$ and $F(\omega ,{\mathbf{n}},{\mathbf{n}}_{{\mathbf{0}}} )$ are the mean values expressed in terms of electron density $\rho ({\mathbf{r}})$ in the form:

2 $$G(\omega ,{\mathbf{n}},{\mathbf{n}}_{{\mathbf{0}}} ) = \frac{1}{{N_{e} }}\int {\rho ({\mathbf{r}})} \left| {{\mathbf{f}}({\mathbf{r}})} \right|^{2} d{\mathbf{r}},\;\;F(\omega ,{\mathbf{n}},{\mathbf{n}}_{{\mathbf{0}}} ) = \frac{1}{{N_{e}^{2} }}\left| {\int {\rho ({\mathbf{r}})} {\mathbf{f}}({\mathbf{r}})e^{{ - i{\mathbf{kr}}}} d{\mathbf{r}}} \right|^{2} ,$$

where $${\mathbf{k}} = {\mathbf{n}}\omega /c$$, $${\mathbf{f(r}}_{a} {\mathbf{)}} = \left[ {{\tilde{\mathbf{E}}}(\omega ) \times {\mathbf{n}}} \right]$$, $$\tilde{E}(\omega ) = \int\limits_{ - \infty }^{ + \infty } {\left( {{\mathbf{E}}({\mathbf{r}}_{a,e} ,t) - \frac{1}{2}\nabla_{a} \left( {\frac{{{\mathbf{E}}({\mathbf{r}}_{a,e} ,t)}}{c}} \right)^{2} } \right)e^{i\omega t} dt} ,$$ where $$\nabla_{a,e} = {\raise0.7ex\hbox{$\partial $} \!\mathord{\left/ {\vphantom {\partial {\partial {\mathbf{r}}_{a,e} }}}\right.\kern-\nulldelimiterspace} \!\lower0.7ex\hbox{${\partial {\mathbf{r}}_{a,e} }$}}$$. Also in Eq. (2), $$\delta_{N} ({\mathbf{p}})$$ is a factor that completely determines the geometric arrangement of atoms in the target, which is calculated in a general form as:

3 $$\delta_{N} ({\mathbf{p}}) = \sum\limits_{a,b} {e^{{i{\mathbf{p}}({\mathbf{R}}_{a} - {\mathbf{R}}_{b} )}} } = \left| {\sum\limits_{a} {e^{{i{\mathbf{pR}}_{a} }} } } \right|^{2} .$$

Summation in Eq. (3) is carried out over all atoms of the considered system. We will use the electronic density $\rho ({\mathbf{r}})$ of the Dirac–Hartree–Fock–Slater model, in which $\rho (r) = \frac{{N_{e} }}{4\pi r}\sum\limits_{i = 1}^{3} {A_{i} \alpha_{i}^{2} e^{{ - \alpha_{i} r}} }$, where $A_{i} ,\alpha_{i}$ are the tabular coefficients that determine the electron density in an atom^31^. Using this model, we can find expressions for $G(\omega ,{\mathbf{n}},{\mathbf{n}}_{{\mathbf{0}}} )$ and $F(\omega ,{\mathbf{n}},{\mathbf{n}}_{{\mathbf{0}}} )$ in an analytical form. As a result, we obtain^28^

$$G(\omega ,{\mathbf{n}},{\mathbf{n}}_{{\mathbf{0}}} ) = [{\mathbf{E}}_{0} \times {\mathbf{n}}]^{2} \left| {F_{1} } \right|^{2} + 6\left( {\frac{\omega }{c}} \right)^{2} \left| {F_{2} } \right|^{2} \left( {\frac{{{\mathbf{E}}_{0} }}{c}} \right)^{4} \sum\limits_{i = 1}^{3} {\frac{{A_{i} }}{{\alpha_{i}^{4} }}} [{\mathbf{n}}_{0} \times {\mathbf{n}}]^{2} + 2\left( {\frac{{E_{0} }}{c}} \right)^{2} \sum\limits_{i = 1}^{3} {\frac{{A_{i} }}{{\alpha_{i}^{2} }}} \frac{{[{\mathbf{E}}_{0} \times {\mathbf{n}}]^{2} }}{{c^{2} }}\left| {F_{2} } \right|^{2}-\frac{\omega}{c}({\mathbf{E}}_{0} {\mathbf{n}})({\mathbf{n}}_{0} {\mathbf{n}}) Im\left(F_1 F_2 \right),\;F(\omega ,{\mathbf{n}},{\mathbf{n}}_{{\mathbf{0}}} ) = \frac{1}{{(4\pi )^{2} }}\left| {\sum\limits_{i = 1}^{3} {A_{i} \alpha_{i}^{2} {\mathbf{J}}_{i} (\omega ,{\mathbf{n}},{\mathbf{n}}_{{\mathbf{0}}} )} } \right|^{2} ,$$

4 $${\mathbf{J}}_{i} (\omega ,{\mathbf{n}},{\mathbf{n}}_{{\mathbf{0}}} ) = \frac{{4\pi F_{1} }}{{p^{2} + \alpha_{i}^{2} }}[{\mathbf{E}}_{0} \times {\mathbf{n}}] + \frac{{8\pi iF_{2} }}{{(p^{2} + \alpha_{i}^{2} )^{2} }}\frac{{{\mathbf{\rm E}}_{0} {\mathbf{p}}}}{{c^{2} }}[{\mathbf{E}}_{0} \times {\mathbf{n}}] - \frac{{4\pi iF_{2} }}{{(p^{2} + \alpha_{i}^{2} )^{3} }}\frac{\omega }{c}\left\{ {\left( {\frac{{E_{0} p}}{c}} \right)^{2} - 4\left( {\frac{{{\mathbf{\rm E}}_{0} \cdot {\mathbf{p}}}}{c}} \right)^{2} + \left( {\frac{{\alpha_{i} E_{0} }}{c}} \right)^{2} } \right\}[{\mathbf{n}}_{0} \times {\mathbf{n}}],$$

where ${\mathbf{p}} = \frac{\omega }{c}({\mathbf{n}} - {\mathbf{n}}_{0} ) = {\mathbf{k}} - {\mathbf{k}}_{0}$ has the meaning of a recoil pulse in the scattering of a USP,$F_{1} = \int\limits_{ - \infty }^{\infty } {v(x)} e^{i\omega x} dx,F_{2} = \int\limits_{ - \infty }^{\infty } {v^{2} (x)} e^{i\omega x} dx,$ expression for $v(x)$ determined: ${\mathbf{E}}(t,{\mathbf{r}}) = {\mathbf{E}}_{0} v(x,\gamma ,\omega_{0} ),x = t - {\mathbf{n}}_{0} {\mathbf{r}}/c$,${\mathbf{n}}_{0}$ is a unit vector along USP, $\gamma$ is the spectral width parameter, $\omega_{0}$ is the carrier frequency, i.e.$v(x,\gamma ,\omega_{0} )$ sets the form of the USP.

Further, for complete definiteness of the scattering spectrum, we define the functions $F_{1} (\omega )$ and $F_{2} (\omega )$. For this, we will consider a USP of the Gaussian form:

5 $${\mathbf{E}}\left( {{\mathbf{r}},t} \right) = {\mathbf{E}}_{0} \exp \left( { - \gamma^{2} \left( {t - {\mathbf{n}}_{0} {\mathbf{r}}/c} \right)^{2} } \right)\cos \left( {\omega_{0} t - {\mathbf{k}}_{0} {\mathbf{r}}} \right).$$

In Eq. (5), $$\user2{k}_{0} = \user2{n}_{0} \;{\raise0.7ex\hbox{${\omega _{0} }$} \!\mathord{\left/ {\vphantom {{\omega _{0} } c}}\right.\kern-\nulldelimiterspace} \!\lower0.7ex\hbox{$c$}}$$, and the pulse duration is $$\tau = 1/\gamma$$, where $$\gamma$$ is a damping parameter in a Gaussian pulse. We will assume that the USP is multi-cycle, i.e. $${\raise0.7ex\hbox{${\omega_{0} }$} \!\mathord{\left/ {\vphantom {{\omega_{0} } \gamma }}\right.\kern-\nulldelimiterspace} \!\lower0.7ex\hbox{$\gamma $}} \gg 1$$. In such a high-cycle case, the condition $$\int_{ - \infty }^{\infty } {{\mathbf{E}}({\mathbf{r}},t)dt = 0}$$ will be met. It is usually used in the case of laser light sources. It should be added that the choice of USP in the form of Eq. (5) is not strictly fixed in our theory. The pulse selection can be arbitrary depending on the USP source. The Gaussian pulse is chosen as one of the best known for describing the USP. For example, in^32^, an exact description of a subcycle pulsed beam (SCPB) was found, where, in the case considered in this paper ($${\raise0.7ex\hbox{${\omega_{0} }$} \!\mathord{\left/ {\vphantom {{\omega_{0} } \gamma }}\right.\kern-\nulldelimiterspace} \!\lower0.7ex\hbox{$\gamma $}} \gg 1$$), the solution has the form of a Gaussian pulse. Further, by taking into account only those terms that will make a significant contribution to the scattering, we obtain:

6 $$F_{1} = \frac{\sqrt \pi }{{2\gamma }}e^{{ - \left( {\frac{{\omega - \omega_{0} }}{2\gamma }} \right)^{2} }} ,F_{2} = \frac{\sqrt \pi }{{4\sqrt 2 \gamma }}e^{{ - \left( {\frac{{\omega - 2\omega_{0} }}{2\sqrt 2 \gamma }} \right)^{2} }} .$$

Also, we define the value ${\mathbf{f}}({\mathbf{r}}_{a,e} )$, which contains information about the USP field [see Eqs. (1) and (2)] as:

7 $${\mathbf{f}}({\mathbf{r}}_{a,e} ) = \exp (i{\mathbf{k}}_{0} {\mathbf{r}}_{a,e} )\left\{ {\left( {F_{1} (\omega ) - F_{2} (\omega )\frac{{{\mathbf{E}}_{0} {\mathbf{r}}_{a,e} }}{{c^{2} }}} \right)\left[ {{\mathbf{\rm E}}_{0} \times {\mathbf{n}}} \right] - iF_{2} (\omega )\frac{\omega }{2c}\left( {\frac{{{\mathbf{E}}_{0} {\mathbf{r}}_{a,e} }}{c}} \right)^{2} \left[ {{\mathbf{n}}_{0} \times {\mathbf{n}}} \right]} \right\}$$

From Eqs. (6) and (7) it can be seen that the contribution to the magnetic component is determined by the factor $F_{2}$. This factor becomes significant only with high-power USPs, which can be seen from Eq. (7). In this case, taking into account the magnetic component will lead to the appearance of the second harmonic, which is clearly seen from the second function in Eq. (6). The line width at the fundamental and second harmonic is determined by the parameter $\gamma$. It should be added that the magnetic component in the scattering of USPs is usually not considered in diffraction analysis, on the basis that its contribution is too small. In fact, if high-power USPs are used, the contribution of the magnetic component can be significant and even comparable with the contribution of the fundamental harmonic. Let us estimate at what power of USP the magnetic component can already be taken into account. In the atomic units in Eq. (7), we can assume that ${\mathbf{r}}_{a,e} \sim 1$. As a result, we must estimate two dimensionless parameters $\frac{{E_{0} }}{{c^{2} }}$ and $\frac{{\omega_{0} }}{c}\left( {\frac{{E_{0} }}{{c^{2} }}} \right)^{2}$. These relations show the magnitude of the magnetic component contribution of the of the USP during its scattering. Modern USP sources can generate radiation intensities $I = cE_{0}^{2} /8\pi \approx 10^{22} W/cm^{2}$ and even greater (e.g.^3^), which correspond to $E_{0} \approx 10^{12} V/cm$ and above. In atomic units, these correspond to $E_{0} \sim 10^{3}$ and above (the atomic unit of field strength $E_{a} = 5.14 \times 10^{9} \;{\text{V}}/{\text{cm}}$). Consider the first parameter $\frac{{E_{0} }}{{c^{2} }}$, where in atomic units $\frac{{E_{0} }}{{c^{2} }} \approx \frac{{E_{0} }}{{137^{2} }}$. In this case, it is seen that for $E_{0} \sim 10^{3}$, the parameter $\frac{{E_{0} }}{{137^{2} }}$ may be significant $\frac{{E_{0} }}{{137^{2} }} \sim 1/10$ and even greater than this value for $E_{0} > 10^{3}$. Consider the second parameter $\frac{{\omega_{0} }}{c}\left( {\frac{{E_{0} }}{{c^{2} }}} \right)^{2}$, which differs from the first factor $\frac{{\omega_{0} }}{c}$. This parameter can be even larger than the first when $\frac{{\omega_{0} }}{c} > 1$ and make a greater contribution. In other words, the magnetic component of USPs can be significant for powerful USP sources.

From Eq. (1) it can be seen that the diffraction pattern is determined mainly by the expression $\delta_{N} ({\mathbf{p}})F(\omega ,{\mathbf{n}},{\mathbf{n}}_{{\mathbf{0}}} )$, which is obvious since the factor $\delta_{N} ({\mathbf{p}})$ determines the geometric arrangement of atoms in the target. It is also seen from Eqs. (1) and (3) that in the case of a polyatomic system, the main part in the scattering spectrum is introduced by the last term in Eq. (1), since the maximum value $\delta_{N}^{\max } ({\mathbf{p}}) = N_{a}^{2}$. At present, it is technically possible to conduct research not only on polyatomic systems, but also on systems where the number of atoms is not large. In this case, we must take into account not only the last term in Eq. (1), but also all the terms in full. In general, Eq. (1) contains both incoherent and coherent parts of the spectrum. In other words, the scattering spectrum can be $\propto N_{e} N_{a}$ for the incoherent part of the spectrum. This case corresponds to scattering of USP by atomic electrons independently of each other. In the case of the coherent part, the scattering spectrum $\propto N^{2}_{e} N^{2}_{a}$. This case corresponds to the scattering of the USP by atomic electrons together.

## Diffraction analysis taking into account the magnetic component of the USP

In this section, using specific examples, we show the effect of the magnetic component of USPs on the scattering spectra. By taking into account the magnetic component of the USP, we also show that the diffraction pattern provides enough information to enable the use of the second harmonic for the diffraction analysis of matter. First, we will look at 2D materials—a group of rings on a plane (or plane group of rings (PGR)), and graphene, and then 3D materials such as a nanotube and a hybrid system consisting of nanotubes.

**Figure 1:**
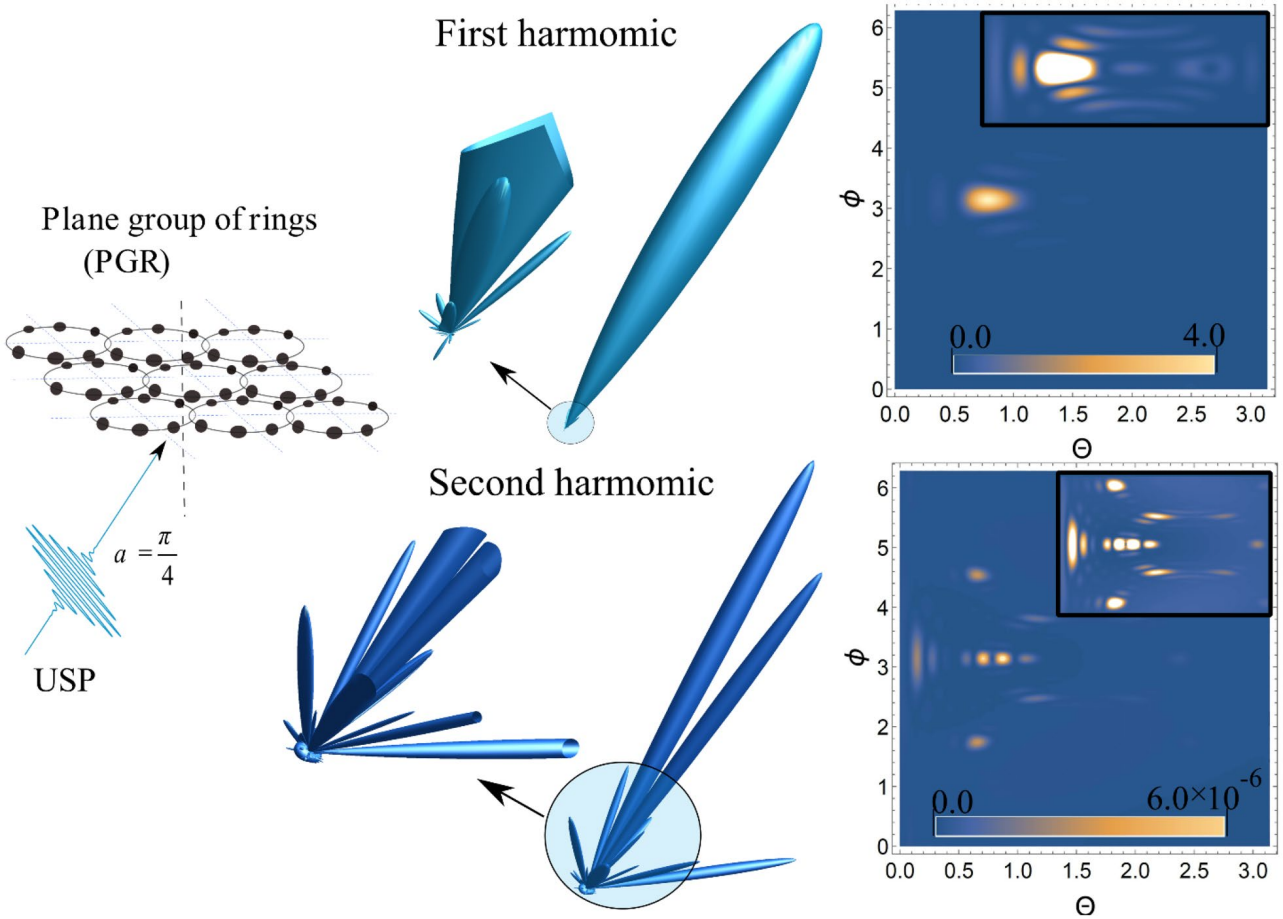
3D radiation pattern of the scattering spectra USP [see Eq. (1)] and the contour plot for the spectra, where $\theta$ and $\phi$ are angles (in radians) with a spherical coordinate system, i.e. polar and azimuth angles, respectively. Inserts in contour plots show an enlarged (more contrasting) scatter spectrum. The first harmonic is in the figures above, and the second harmonic is below. A USP was selected with a frequency $\omega_{0}$=100 au, a pulse duration $\gamma$ corresponding to 43 as and amplitude $E_{0}$=1000 au. For the plane group of rings, the number of atoms in the ring is N = 6, the radius of the ring is R = 4 au, the number of grid nodes is 5 × 5 au, (25 rings), and the distance between the centers of adjacent rings is d = 7 au. The angle of incidence $\alpha$between ${\mathbf{n}}_{0}$ and the z axis was selected as$\alpha = \pi /4$.

Consider a group of rings consisting of $N_{n}$ identical atoms on the n*th* ring, located along the circumference of the ring with radius $R_{n}$, equidistant from each other, see Fig. 1. We introduce a rectangular coordinate system so that the origin of the coordinate system is in the center of the ring, and the x, y axes lie in the plane of the ring. As a result, the factor $\delta_{N} ({\mathbf{p}})$ for such a system will be in the form^28^:

8 $$\delta_{N} ({\mathbf{p}}) = \left( {\sum\limits_{n = 1}^{M} {N_{n} J_{0} \left( {R_{n} \left| {{\mathbf{p}} \times {\mathbf{k}}} \right|} \right)} } \right)^{2} \prod\limits_{i = 1}^{2} {\left( {\frac{{\sin \left[ {L_{i} {\mathbf{pd}}_{i} /2} \right]}}{{\sin \left[ {{\mathbf{pd}}_{i} /2} \right]}}} \right)^{2} } ,$$

where M is the number of rings, $J_{0}$ is the zero-order Bessel function, $N_{n}$ is the number of atoms in a ring with radius $R_{n}$, $L_{1} ,L_{2}$ are the numbers of nodes on the x, y axes, respectively, and $d_{1} ,d_{2}$ are the lattice periods.

Using Eqs. (1) and (8) we now calculate the spectra for the scattering of USPs on such a system for different spatial locations of the detector, as specified by the angles Θ and ϕ. An example of such a calculation is shown in Fig. 1: 3D plots show the spatial distribution of the intensity of the scattered USP; 2D plots show the same distribution, but in the form of contour plots, which demonstrate the magnitude of the scattering spectra of the USPs. It can be seen that scattering at the first harmonic, and hence without taking into account the magnetic field, has certain diffraction maxima that are characteristic of this system. Of these diffraction maxima, there is one main maximum, the intensity of which is significantly higher than the others. This maximum has a clearly pronounced direction in the direction of the falling pulse **n**_**0**_. If we consider scattering at the second harmonic, i.e. taking into account the magnetic component of the USP, we will also see the diffraction maxima. There are noticeably more of these maxima than at the first harmonic, and they are more pronounced. In other words, the diffraction pattern at the second harmonic is more informative. In contrast to the first harmonic, the direction of the scattered USP at the second harmonic differs markedly from the direction **n**_**0**_ of the falling pulse. Both parts of the spectrum are sensitive to the number of ring atoms: the greater the number of atoms, the smaller the solid angle of the diffraction peak.

Thus, most of the radiation falls on the carrier frequency ω_0._ With an increase in the radius of the system and the number of atoms in it, the spectrum degenerates into a delta function.

**Figure 2:**
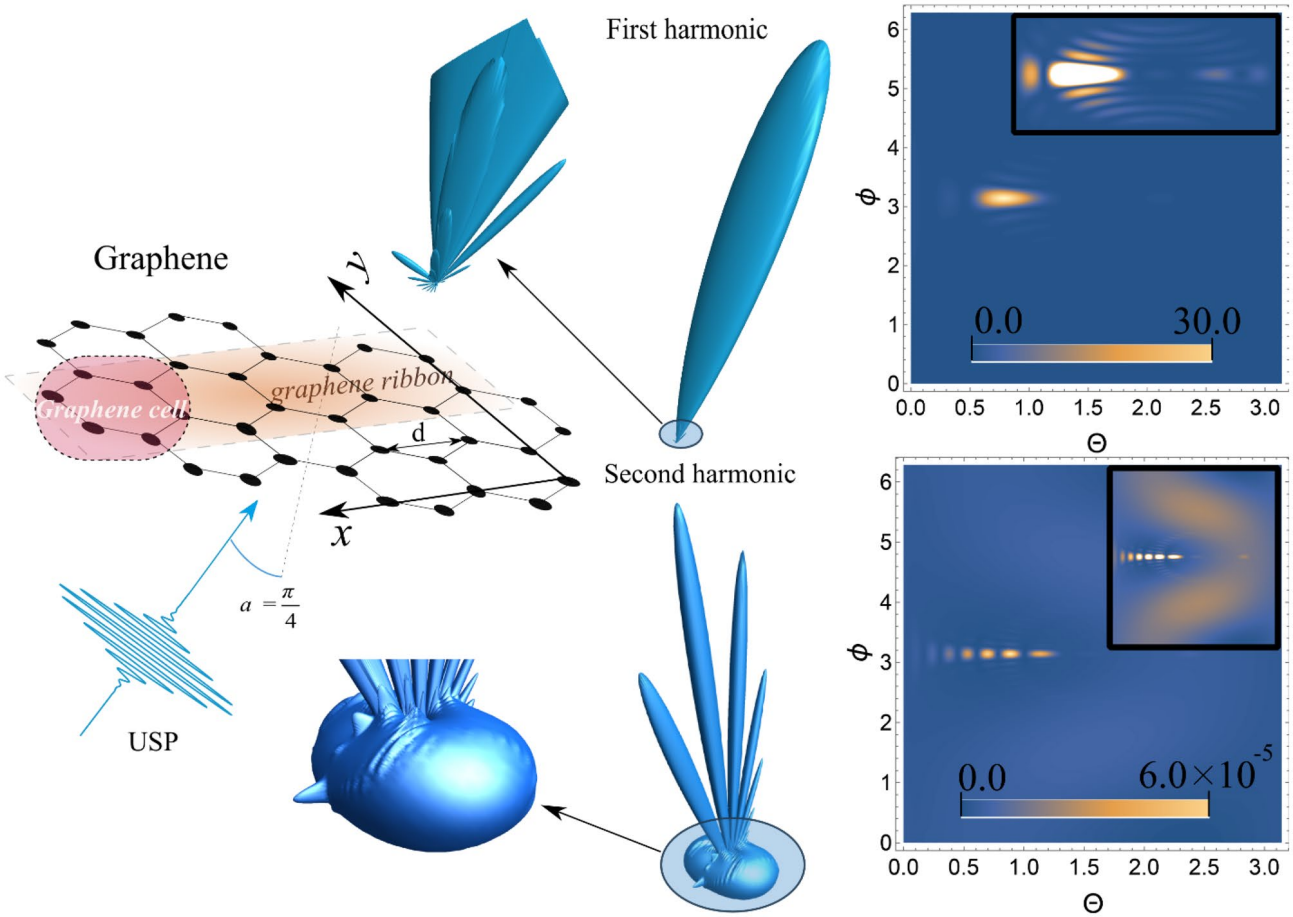
3D radiation pattern of the scattering spectra USP [see Eq. (1)] and the contour plot for the spectra, where $\theta$ and $\phi$ are angles (in radians) with a spherical coordinate system, i.e. polar and azimuth angles, respectively. Inserts in contour plots show an enlarged (more contrasting) scatter spectrum. The first harmonic is in the figures above, and the second harmonic is below. A USP was selected with a frequency $\omega_{0}$=100 au, a pulse duration $\gamma$ corresponding to 43 as and amplitude $E_{0}$= 1000 au. The numbers of the graphene ribbons and cells are $L = 10$, $N = 10$, respectively The angle of incidence$\alpha$between ${\mathbf{n}}_{0}$ and the z axis was selected as $\alpha = \pi /4$.

Next, we consider another 2D material: graphene. The factor $\delta_{N} ({\mathbf{p}})$ for graphene was partially calculated in^33^. As a result, the factor $\delta_{N} ({\mathbf{p}})$ can be represented as:

9 $$\delta_{N} ({\mathbf{p}}) = \frac{{4\sin^{2} \left( {\frac{\sqrt 3 }{2}L{\mathbf{pj}}d} \right)}}{{\sin^{2} \left( {\frac{{{\mathbf{pi}}d}}{2}} \right)\sin^{2} \left( {\frac{\sqrt 3 }{2}{\mathbf{pj}}d} \right)}}\left\{ {\cos \left( {\frac{{{\mathbf{pj}}d}}{\sqrt 3 }} \right)\sin \left( {\frac{{{\mathbf{pi}}dN}}{2}} \right) + } \right.\left. {\cos \left( {\frac{{{\mathbf{pj}}d}}{2\sqrt 3 }} \right)\sin \left( {\frac{{{\mathbf{pi}}d(N + 1)}}{2}} \right)} \right\}^{2} ,$$

where $L$ is the number of graphene ribbons, $N$ is the number of the cells in graphene tape, $d$ is the distance between atoms along the *x* axis, and ***i*** and ***j*** are unit vectors along the x and y axes, respectively, see Fig. 2.

Using Eqs. (1) and (9), the spectra were calculated for the scattering of USPs on graphene. An example of such a calculation is shown in Fig. 2; the 3D and 2D graphs are described similarly to those in Fig. 1. Scattering at the first harmonic, and therefore without taking into account the magnetic field, has certain diffraction maxima that are characteristic of this system. It should be noted that the diffraction pattern at the first harmonic is quite close to the diffraction pattern when USPs are scattered by a system of rings (see Fig. 1). In the case of scattering at the second harmonic, the diffraction maxima characteristic of graphene can also be seen. There are noticeably more bright peaks than at the first harmonic, and they are more pronounced. The diffraction pattern presented in the second harmonic scattering spectra among these 2D objects (PGR, graphene) has obvious differences. Therefore, the analysis of the spectra will make it possible to more correctly determine the studied system.

**Figure 3:**
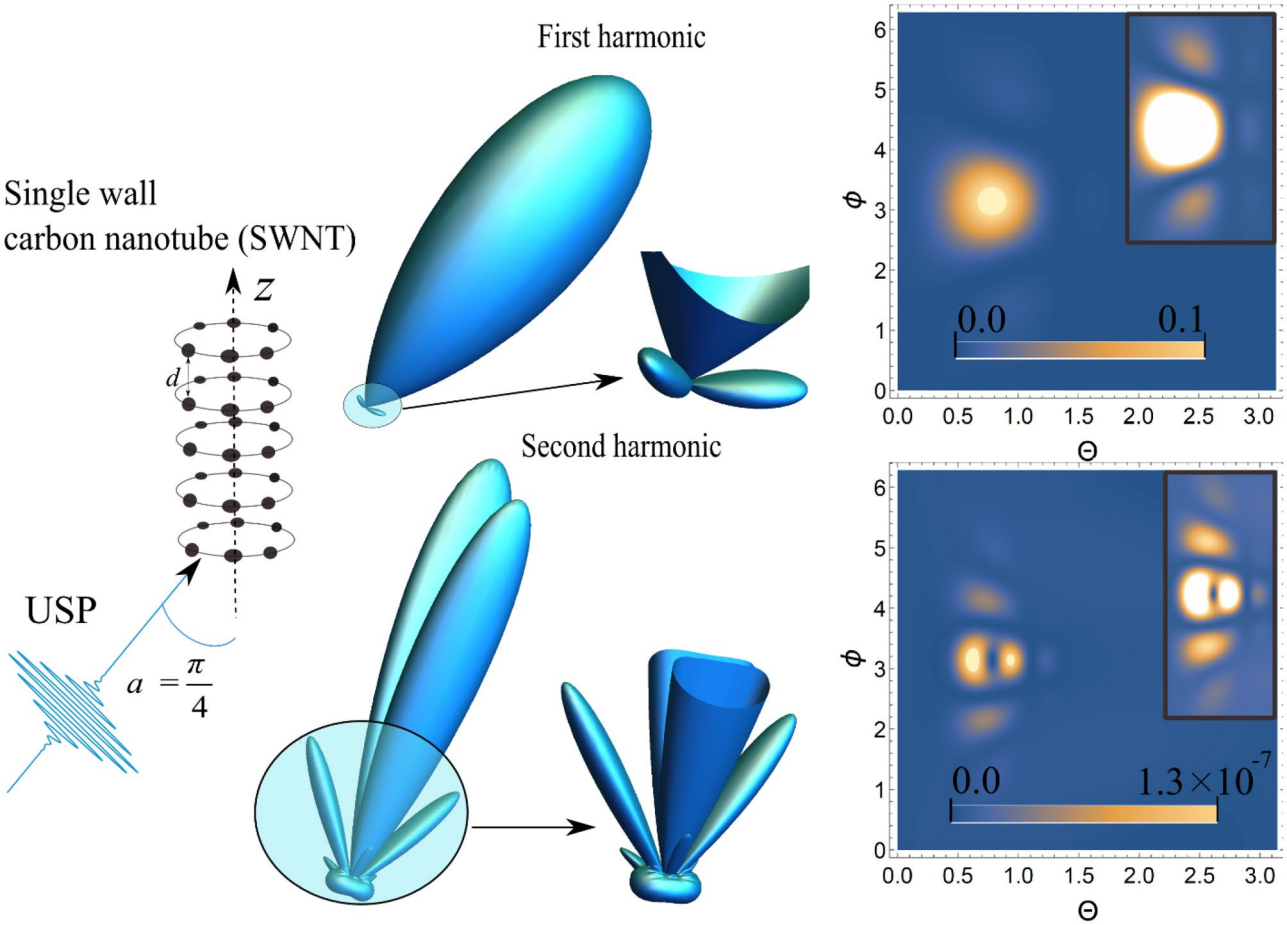
3D radiation pattern of the scattering spectra USP [see Eq. (1)] and the contour plot for the spectra, where $\theta$ and $\phi$ are angles (in radians) with a spherical coordinate system, i.e. polar and azimuth angles, respectively. Inserts in contour plots show an enlarged (more contrasting) scatter spectrum. The first harmonic is in the figures above, and the second harmonic is below. A USP was selected with a frequency $\omega_{0}$=100 au, a pulse duration $\gamma$ corresponding to 43 as. and amplitude $E_{0}$= 1000 au. The number of planes$L = 8$, the distance between planes $d = 2$au, and the ring radius $R = 4$au. The angle of incidence$\alpha$between ${\mathbf{n}}_{0}$ and the z axis was selected as $\alpha = \pi /4$.

We now consider a 3D system in the form of a CNT, in the approximation of an axially symmetric group of rings, see Fig. 3. The factor $\delta_{N} ({\mathbf{p}})$ for such a system was studied in^28^, and as a result:

10 $$\delta_{N} ({\mathbf{p}}) = \left( {\sum\limits_{n = 1}^{M} {N_{n} J_{0} \left( {R_{n} \left| {{\mathbf{p}} \times {\mathbf{k}}} \right|} \right)} } \right)^{2} \left( {\frac{{Sin\left[ {Ld{\mathbf{pk}}/2} \right]}}{{Sin\left[ {{\mathbf{pk}}d/2} \right]}}} \right)^{2} ,$$

where $N_{n}$ is number of atoms in a ring *n* with radius $R_{n}$, L is the number of planes with rings, d is the step between planes, and ${\mathbf{k}}$ is the unit vector directed along the *z* axis. Thus, we can define the analog of both a single layer M = 1 and a multi-layer M > 1 CNT.

Below we demonstrate the scattering spectra of USPs by a CNT for different spatial locations of the detector as specified by the angles Θ and ϕ. An example of such a calculation is shown in Fig. 3; the 3D and 2D graphs are described similarly to those in Fig. 1. It can be seen that the scattering at the first harmonic has certain diffraction maxima that are characteristic of this system; of these, there is one main maximum, the intensity of which is significantly higher than the others. This maximum has a clearly defined direction along the vector **n**_**0**_, i.e. in the direction of the falling pulse. If we consider the scattering at the second harmonic, we will see that there are more maxima than at the first harmonic, and they are more pronounced. The diffraction pattern at the second harmonic carries more information than at the first. The direction of the scattered USP at the second harmonic differs markedly from the direction **n**_**0**_ of the pulse falling, in contrast to the first harmonic. Both parts of the spectrum are sensitive to both the number of ring atoms and the angle of incidence of the pulse. With an increase in the number of atoms, the spectrum acquires a smaller solid angle of the diffraction peak.

**Figure 4:**
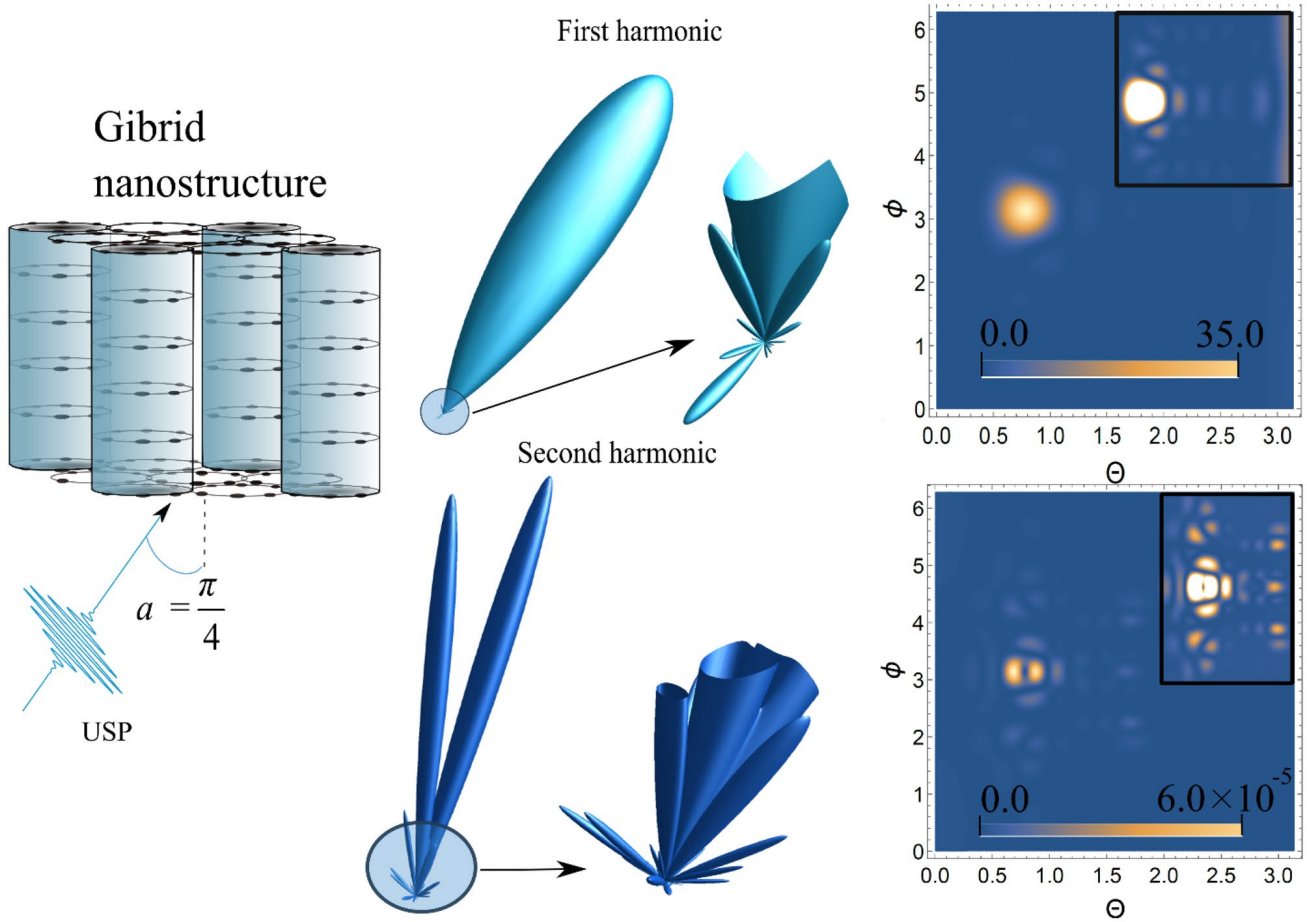
3D radiation pattern of the scattering spectra USP [see Eq. (1)] and the contour plot for the spectra, where $\theta$ and $\phi$ are angles (in radians) with a spherical coordinate system, i.e. polar and azimuth angles, respectively. Inserts in contour plots show an enlarged (more contrasting) scatter spectrum. The first harmonic is in the figures above, and the second harmonic is below. A USP was selected with a frequency $\omega_{0}$=100 au, a pulse duration $\gamma$ corresponding to 43 as and amplitude $E_{0}$= 1000 au. The numbers of nodes $n_{3} = 5$, $n_{1} = n_{2} = 3$, distance $d_{1} = d_{3} = d_{3} = 5$au, number of atoms in a ring $N = 6$, and ring radius $R = 3$au. The angle of incidence$\alpha$between ${\mathbf{n}}_{0}$ and z axis was selected as $\alpha = \pi /4$.

We now consider a composite nanostructure, that of a hybrid system consisting of CNTs and PRG, see Fig. 4. In such a system, CNTs connect the two PRG planes discussed above. We will consider the case where CNTs alternate through one ring in the PGR plane (like a chessboard). The factor for such a system $\delta_{N} ({\mathbf{p}})$, has not yet been studied, but is simple to obtain from the previous cases for PGR and a CNT:

11 $$\delta_{N} ({\mathbf{p}}) = \left( {\sum\limits_{n = 1}^{M} {N_{n} J_{0} \left( {R_{n} \left| {{\mathbf{p}} \times {\mathbf{k}}} \right|} \right)} } \right)^{2} \left| {\alpha_{1} \alpha_{2} \left( {\frac{{1 - e^{{i{\mathbf{pk}}d_{3} n_{3} }} }}{{1 - e^{{i{\mathbf{pk}}d_{3} }} }}} \right) + \beta_{1} \beta_{2} \left( {1 + e^{{i{\mathbf{pk}}d_{3} (n_{3} - 1)}} } \right)} \right|^{2} ,\alpha_{k} = \frac{{1 - e^{{i{\mathbf{pd}}_{k} (1 + [n_{k} ])}} }}{{1 - e^{{2i{\mathbf{pd}}_{k} }} }},\beta_{k} = e^{{i{\mathbf{pd}}_{k} }} \frac{{1 - e^{{i{\mathbf{pd}}_{k} [n_{k}^{^{\prime}} ]}} }}{{1 - e^{{2i{\mathbf{pd}}_{k} }} }}$$

where $d_{1}$ and $d_{2}$ are the periods of the lattice in the centers of which the rings are located along the *x* and *y* axes, respectively (*x* and *y* are in the plane of the rings), $n_{3}$ is the number of nodes along the *z* axis, $[n_{1} ]$ and $[n_{2} ]$ are the numbers of odd atoms along the *x* and *y* axis, respectively, and $[n_{1}^{^{\prime}} ]$ and $[n_{2}^{^{\prime}} ]$ are the numbers of even atoms along the *x* and *y* axes, respectively.

Below, we present the calculations of the scattering spectra of USPs on a composite carbon system. An example of such a calculation is shown in Fig. 4; 3D and 2D graphs are described similarly to those in Fig. 1. The scattering at the first harmonic has certain diffraction maxima that are characteristic of this system; of these there is one main maximum, the intensity of which is significantly higher than the others. This maximum has a clearly defined direction along the vector **n**_**0**_, i.e. in the direction of the falling pulse. If we consider the scattering at the second harmonic, we will see that there are more maxima than at the first harmonic, and they are more pronounced. The direction of the scattered USP at the second harmonic differs markedly from the direction n_0_ of the falling pulse. Both parts of the spectrum are sensitive to the number of ring atoms.

It can be seen from the presented plots that taking into account the magnetic component of the USP, which leads to the appearance of the second harmonic in the scattering spectrum, i.e. makes a noticeable contribution to the overall diffraction pattern. At the second harmonic, this pattern has more than one clearly pronounced diffraction maximum, in contrast to the first harmonic. It should be added that the absolute value of the spectrum at the second harmonic can be significantly greater if a larger value is chosen $E_{0}$ и $\omega_{0}$.

## Discussion and conclusion

We have shown that by using Eq. (1), it is possible to calculate the scattering spectra of USPs with allowance for the magnetic component of the electromagnetic field. The magnetic component leads to the generation of the second harmonic, which can be used for X-ray diffraction analysis of the substance. The above examples demonstrate that the second harmonic leads to a diffraction pattern that has more maxima and is more pronounced than the diffraction pattern at the first harmonic. We should add that this important result is applicable not only to the cases considered above, but is a fundamental consequence of the USP scattering with allowance for the second harmonic and is general. We will show this using Eq. (3). Since the factor is responsible for the geometric arrangement of atoms in the system, it is responsible for the diffraction pattern. The factor can be presented in another form:

12 $$\delta_{N} ({\mathbf{p}}) = \left| {\int {\rho ({\mathbf{R}})e^{{i{\mathbf{pR}}}} d^{3} {\mathbf{R}}} } \right|^{2} ,\rho ({\mathbf{R}}) = \sum\limits_{a} {\delta ({\mathbf{R}} - {\mathbf{R}}_{a} )} ,$$

where $$\delta ({\mathbf{R}} - {\mathbf{R}}_{a} )$$ is the Dirac delta function. Since we assume that $${\raise0.7ex\hbox{${\omega_{0} }$} \!\mathord{\left/ {\vphantom {{\omega_{0} } \gamma }}\right.\kern-\nulldelimiterspace} \!\lower0.7ex\hbox{$\gamma $}} \gg 1$$, then in this case the value $$dW/d\Omega_{{\mathbf{k}}}$$[see Eq. (1)], when integrated over frequencies, will be concentrated near the two frequencies $$\omega_{0}$$ and $$2\omega_{0}$$. In this case the vector $${\mathbf{p}}$$ is also concentrated near two frequencies $${\mathbf{p}}_{1} = \omega_{0} /c({\mathbf{n}} - {\mathbf{n}}_{0} )$$ and $${\mathbf{p}}_{2} = 2\omega_{0} /c({\mathbf{n}} - {\mathbf{n}}_{0} )$$. This, in turn, leads to the fact that the factor $$\delta_{N} ({\mathbf{p}})$$ is also situated near the first $$\omega_{0}$$ and $$2\omega_{0}$$ harmonics. Since $${\mathbf{p}}_{2} = 2{\mathbf{p}}_{1}$$, then the number of oscillations as a result of integration in $$\int {\rho ({\mathbf{R}})e^{{i{\mathbf{p}}_{2} {\mathbf{R}}}} d^{3} R}$$ will be more than $$\int {\rho ({\mathbf{R}})e^{{i{\mathbf{p}}_{1} {\mathbf{R}}}} d^{3} R}$$, leading to the fact that there will be more diffraction maxima at the second harmonic. For example, this can be seen directly from the factors $$\delta_{N} ({\mathbf{p}})$$ in Eqs. (8)–(11), and is easily shown in the case of a rectangular lattice, where the delta factor (see e.g.^34^) is determined:

13 $$\delta_{N} ({\mathbf{p}}) = \prod\limits_{k = 1}^{3} {\frac{{\mathop {sin}\nolimits^{2} ({\mathbf{pd}}_{k} N_{k} /2)}}{{\mathop {sin}\nolimits^{2} ({\mathbf{pd}}_{k} /2)}}} ,$$

where ${\varvec{d}}_{1} ,{\varvec{d}}_{2} ,{\varvec{d}}_{3}$ are the interatomic distances and directions along the *x, y, z* axes, respectively, and ${\rm N}_{1} ,{\rm N}_{2} ,{\rm N}_{3}$ are the numbers of atoms along selected *x, y, z* axes, respectively. With a sufficiently large number of atoms in the system, Eq. (13) shows that the peaks will be at: $\omega_{0} /c(\user2{n - n}_{0} ){\varvec{d}}_{1} = 2\pi n,\omega_{0} /c(\user2{n - n}_{0} ){\varvec{d}}_{2} = 2\pi m,\omega_{0} /c(\user2{n - n}_{0} ){\varvec{d}}_{3} = 2\pi k$, where $n,m,k = 0,1,2,...$ are integers. This is the Laue condition if we assume that the scattering occurs at the fundamental frequency $\omega_{0}$. If we consider scattering by the second harmonic, then we obtain a certain analog of the Laue condition in the form:$\omega_{0} /c({\varvec{n}} - {\varvec{n}}_{0} ){\varvec{d}}_{1} = \pi n,\omega_{0} /c({\varvec{n}} - {\varvec{n}}_{0} ){\varvec{d}}_{2} = \pi m,\omega_{0} /c({\varvec{n}} - {\varvec{n}}_{0} ){\varvec{d}}_{3} = \pi k$. Comparing these two expressions, it can be seen that the second harmonic does indeed have a large number of diffraction peaks.

As a result, we obtain two diffraction patterns on the fundamental and second harmonics, both of which were derived from the same USP and on a given polyatomic system. As a result, diffraction analysis can be carried out by studying two diffraction patterns at once, which allows you to get more details about the research object. It should be added that the approach developed here, as also shown in^28^, is a special case corresponding to well-known techniques in X-ray and diffraction analysis.

The theory developed here takes into account aspects of the interaction of USPs with matter. Note that using Eq. (1), both stationary and dynamic systems can be studied. In this case, it is necessary to replace in Eq. (1) the density $\rho ({\mathbf{r}})$ by $\rho ({\mathbf{r}},t)$, where is the moment in time at which the USP acts on the system under study^19^. These dynamic systems include complex molecules, including biomolecules, where bonds are broken or formed, as well as peptides and biological systems within which there is charge migration.

It should be added that the sizes of the objects that were considered here and which we are interested in are less than the extinction length in dynamic diffraction. This means that dynamic diffraction can be neglected. Although the diffraction at the second harmonic will also be on larger objects, where dynamic diffraction cannot be neglected. How scattering at the second harmonic will affect the effects in the dynamic theory of diffraction is an interesting question and can be investigated in the future.

Thus, the method presented here is sensitive to the geometry of nano-objects and may soon supplement X-ray structural analysis. The authors expect their work to become a starting point for experimental research aimed at studying biomolecules and various dynamic systems, as well as for detecting defects in 2D and 3D nanosystems.
